# Two-Way Efforts Between the Organization and Employees: Impact Mechanism of a High-Commitment Human Resource System on Proactive Customer Service Performance

**DOI:** 10.3390/bs15030321

**Published:** 2025-03-06

**Authors:** Dexia Zang, Boyi Lyu

**Affiliations:** Business School, Hohai University, Nanjing 211100, China; zangdexia@163.com

**Keywords:** proactive customer service performance, high-commitment human resource system, mission valence, work meaning, serial mediation model

## Abstract

Service quality forms the foundation of customer experience value and is a key competitive edge for service-oriented organizations. In response to increasingly diverse service demands, proactive customer service performance (PCSP), which can improve service experience, has captured the attention of researchers and managers. While research on factors influencing PCSP is plentiful, there is a noticeable gap in discussions around organizational-level factors, especially concerning a high-commitment human resource system (HCHRS) designed to enhance positive relationships between organizations and employees. This study collected data from frontline service employees in China and their managers through a questionnaire survey grounded in self-determination theory (SDT), conservation of resources theory (COR), and social exchange theory (SET) and used hierarchical multiple regression and a mediation effect test to investigate the impact mechanism of the HCHRS on PCSP. This study reveals that the HCHRS directly influences PCSP and positively impacts it by bolstering mission valence (MV) and work meaning (WM). Furthermore, MV and WM serve as serial mediators of the process through which HCHRS affects PCSP. This study enriches research on the antecedent mechanisms of PCSP and offers valuable insights for management practices.

## 1. Introduction

Service quality is the basis of customer satisfaction, and enhancing it is essential for building and improving the competitiveness of service industry organizations at the micro level. At the macro level, the service industry must enhance its capacity to better serve people’s lives and significantly cultivate a new productive force within the industry. Improvement in service quality cannot be separated from frontline employees.

In the context of increasing uncertainty in the external environment and increasingly diversified, refined, and complex customer needs, frontline service employees work as the primary customer-facing window (Cheng et al., 2020; Rod et al., 2016) and must be able to respond to unexpected customer needs. A proactive customer service performance (PCSP) that goes beyond standard procedures is increasingly important for enhancing customer satisfaction (Bitner et al., 1990; Parker et al., 2010). Therefore, one-size-fits-all, standardized, and passive services can no longer adapt to the ever-changing market environment. In particular, when uncertainty in the work environment increases, and it becomes difficult to establish standardized processes, employees’ adaptability and initiative become increasingly important (Griffin et al., 2007). As a result, the spontaneous generation of PCSP by employees has captured the attention of managers. Their goal is to offer services that cater to customers’ emotional needs, creating surprising or moving experiences that boost customer satisfaction and, in turn, enhance the competitiveness of service organizations.

PCSP is an employee’s initiative to provide services beyond the standard service process and requires three properties: self-start, long-term orientation, and persistence (Rank et al., 2007). Employees spontaneously generate PCSP and can provide customers with more personalized or emotionally valuable services by anticipating and meeting their potential needs. This type of service does not directly benefit the employee in return. Still, it promotes positive interaction between the employee and the customer, thus improving the performance of the individual employee and the organization as a whole. Existing research on PCSP involves many service industry segments (Kan et al., 2024; Al Shaer et al., 2023; Siami et al., 2022; Raub & Liao, 2012; Söderlund, 2018) and has received attention from researchers in multiple countries. Since Rank et al. (2007) first identified the concept of PCSP and developed a PCSP measurement scale, a rich and diverse body of research has been conducted; however, existing studies still have certain shortcomings.

First, such studies mainly focus on the influences of leadership, customers, and organizational climate, such as abusive supervision (Zang et al., 2021), ethical leadership (S. Q. Song et al., 2023), customer support (H. Zhang et al., 2021), and workplace ostracism (Zhu et al., 2017). However, these factors may be short-term and unstable. If the leaders, colleagues, or customers that employees face on a daily basis are friendly, employees’ PCSP may be produced smoothly. If a few unsatisfactory leaders or customers exist, the enthusiasm of employees’ PCSP may be difficult to sustain. Systematic and institutionalized factors are more stable and will not be easily changed by human factors. The new generation of employees has higher requirements for work treatment and personal career development and pays more attention to the value and meaning of work. Additionally, the traditional human resource system (HR system), such as a control-oriented or cost-reduction HR system, often fails to meet these needs (M. Song et al., 2018). A high-commitment human resource system (HCHRS) focuses on the rights and interests and development of employees as an internal interest group, ultimately reflecting the organization’s social responsibility fulfillment, which helps to enhance employees’ willingness to take the initiative to service work (G. Zhang & Liu, 2019). However, discussions on the influence of an organization’s human resource management, particularly the impact of the HR system, remain limited, and room for expanding research perspectives still exists.

Second, from the organization’s perspective, the ultimate goal of the HCHRS, which is beneficial to employees, is to achieve a “two-way effort” between the organization and employees, forming a positive and sustainable exchange relationship between the organization and its employees. The HCHRS improves employees’ recognition of the organization’s mission, enhancing their job satisfaction. This ultimately increases employees’ work initiative, leading them to give back to the organization, thereby improving its performance; consequently, organizational performance improves. However, this perspective has not received enough attention in existing research.

Finally, from this perspective, mission valence (MV) and work meaning (WM) may play an important role in the process of HCHRS influencing PCSP. MV can measure employees’ recognition and integration of the organization’s mission into their work, and WM can measure employees’ satisfaction with their work. However, existing studies do not focus on the role of MV in influencing PCSP, with less attention paid to its antecedents. Although some have verified the impact of WM on employees’ extra-role behavior and proactive behavior (Z. Ji & Xu, 2023; Johnson & Jiang, 2017; Kosfeld et al., 2017), to the best of our knowledge, no study has focused more specifically on the impact of WM on PCSP. The existing literature has paid less attention to the factors influencing WM at the organizational level as well.

Aiming to address the limitations of existing studies, this study synthesized self-determination theory (SDT), social exchange theory (SET), and conservation of resource theory (COR) and introduced MV and WM as mediating variables in the process of HCHRS influencing PCSP to further explore the influence mechanism of HCHRS on PCSP. The purpose of this study is to enrich the research on the influencing factors of PCSP and inform service industry management practices.

## 2. Research Hypotheses

### 2.1. High-Commitment Human Resource System and Proactive Customer Service Performance

A growing body of research suggests that HR systems can impact individual employees, teams, and entire organizations (Gong et al., 2025; X. Chen & Xiao, 2020). The HCHRS is an HR system in which an organization establishes a psychological connection with its employees to shape their behavioral patterns and emphasizes establishing a mutual long-term exchange relationship (Collins & Smith, 2006). In strategic human resource management research, the HCHRS has been shown to correlate positively with organizational performance (McClean & Collins, 2011). It has been found that HCHRS also positively affects service organization (Batt, 2002).

X. Chen and Xiao (2020) categorized the HCHRS into ability enhancement (e.g., extensive training), motivation enhancement (e.g., developmental performance appraisal, good salary level), and more opportunities (e.g., flexible job content, employees have the opportunity to participate in organizational decision-making, and information sharing within the organization) according to the framework of “ability–motivation–opportunity” (AMO). This study also adopts the AMO framework and defines the HCHRS as a human resource management mode that establishes a psychological link between the organization and employees to guide employee attitudes and behaviors.

PCSP refers to the initiative of employees to provide services beyond the standard service process and their own performance within their roles. It includes the three properties of being self-started, long-term-oriented, and persistent (Rank et al., 2007). First, employees must develop self-starting behaviors, such as providing services that go beyond customer or supervisor requirements. Secondly, employees must adopt a long-term perspective by anticipating potential customer needs and collaborating with coworkers to fulfill service requirements. This approach promotes positive interactions between employees and customers. Finally, employees’ service needs to be persistent. This includes fulfilling the promised special service, communicating with coworkers about interfacing work so that customers can transition smoothly to the next phase of service, and actively asking customers if they are satisfied with the service. In this study, the concept of PCSP is defined as the self-started, long-term-oriented, and persistent service behavior of frontline service employees after accurately predicting the potential needs of customers.

SDT has undergone a long period of verification and development since 1985, and the theoretical construction is very mature (Deci & Ryan, 1985; Zhao et al., 2016). According to SDT, employees’ proactive behaviors are driven by fulfilling three fundamental needs. The first of these is autonomy; for example, employees enjoy the full trust of the organization or the freedom to explore the content and methods of their own work. The second is perceived competence, that is, employees have full confidence in their ability to complete their work tasks satisfactorily. The last fundamental need is relatedness, which reflects employees’ sense of belonging in the organization.

SDT has been favored by many studies on PCSP. These studies noted that a relaxed and pleasant workplace atmosphere, close relationships with colleagues and leaders, giving employees more work autonomy, and meeting employees’ needs for recognition can all help meet employees’ needs for autonomy and perceived competence and enhance their sense of belonging, thereby increasing their PCSP (Wu et al., 2020; L. Ji et al., 2022; Ma & Hu, 2023). The HCHRS empowers employees with broader job content and more job autonomy through process-based performance appraisal, implementation of internal rotation, and employee participation in decision-making. This reflects the organization’s trust in frontline service employees and enhances their autonomy. Through careful recruitment, adequate training, implementation of internal rotation, and other measures, the HCHRS strengthens employees’ perception of their own competence within the organization and ensures that frontline service employees are equipped with the skills necessary to cope with their work, i.e., the perception of competence. The HCHRS also promotes egalitarianism in income, status, and culture, information-sharing between the organization’s management and employees, and competitive remuneration systems. These opportunities can satisfy frontline service employees’ needs for a sense of belonging to the organization from both material and spiritual perspectives.

In summary, the HCHRS can contribute to the formation of PCSP in frontline service employees by satisfying their work autonomy, perceived competence, and relationship needs. Therefore, this study proposes Hypothesis 1:

**H1.**  *HCHRS positively influences frontline service employees’ PCSP*.

### 2.2. The Mediating Role of Mission Valence

The connotations of MV can be sorted from the perspective of individual perception and organizational effectiveness (Yu & Zhang, 2023). The individual perception perspective focuses on how employees personally perceive the organization’s mission, highlighting its attractiveness as seen through the eyes of the employees (Rainey & Steinbauer, 1999). The organizational effectiveness perspective argues that MV, through a clear and attractive vision of organizational goals, shows employees how their work contributes to achieving organizational goals and benefits society. This can stimulate employees’ sense of responsibility and guide them to complete their tasks efficiently, even after external rewards have been kept in check (Wright et al., 2012; Yu & Zhang, 2023). According to stakeholder theory, employees are important stakeholders in the organizational management process, and MV can reflect the degree of internal consistency between individuals and an organization’s long-term goals and values (Guerrero & Chênevert, 2021). MV directly affects employees’ work behaviors (Yuan et al., 2022) and is an important factor in achieving individual development and enhancing organizational performance (Yu & Zhang, 2023). This study integrates the individual perception and organizational effectiveness perspectives to define MV as frontline service employees’ perception of the importance of the organizational mission. This importance influences their willingness to actively integrate into the organizational mission and participate in achieving organizational goals.

SET is a classic theory in sociological research (Blau, 2017). The theory proposes the principle of reciprocity, which means that when individuals are treated well by others, they will give equal or greater returns, and the two parties will thus form a reciprocal exchange relationship. Owing to the universality of social exchange and the principle of reciprocity, SET has been widely used in organizational management research. Existing studies have established that the good deeds of an organization to its employees can be exchanged for employees’ rewards. The more a service organization implements distributive justice and procedural justice, the more it can motivate individuals in the organization to repay the organization through PCSP (Abuelhassan & AlGassim, 2022). Lau et al. (2017) highlighted that shaping a positive ethical work climate can encourage employees to give back and spread virtues and have a positive impact on employees’ emotional commitment and PCSP.

HCHRS creates an environment of mutual benefit between the organization and its employees, which can lead employees to generate positive and beneficial responses to the organization (McClean & Collins, 2011). The literature has noted that implementing HCHRS encourages individuals to prioritize the organization’s interests over theirs (Tsui et al., 1995). According to SET (Blau, 2017), organizations invest in their employees through the HCHRS to improve their overall performance and build long-term relationships. As stakeholders in the business process, employees recognize that the organization fulfills its responsibilities toward them. They also believe that the organization’s mission benefits society. To reciprocate the organization’s goodwill, employees will become more positively integrated into the organization’s mission and demonstrate a higher willingness to contribute to the realization of organizational goals.

COR is the most common theory in the study of PCSP. This theory posits that “resources” are the total ability of an individual to meet their core needs. Owing to the valuable nature of resources, individuals strive to obtain and protect their own resources. When they realize the loss of resources caused by stressors, they will experience psychological stress, reduce resource investment in work, and reduce work initiative. When employees have abundant resources, they will invest more resources in their work, thus making their work performance more proactive.

Frontline service work involves emotional labor, and emotional and psychological resources are important (Cheng et al., 2020). Socially responsible human resource management provides employees with more external resource support, which strengthens their identification with the organization and stimulates their PCSP (G. Zhang & Liu, 2019). It has been found that the more appealing the organizational mission is to employees, the more proactive their performance is (Rainey & Steinbauer, 1999). Additionally, increased MV prompts employees to implement more extra-role behaviors (Caillier, 2016). Yuan et al. (2022) have also focused on the role of MV in mediating the relationship between public service motivation and the proactive behavior of public servants. Therefore, according to COR (Hobfoll, 1989), the adequacy of work resources improves the affective state of frontline service employees, reduces the likelihood of emotional exhaustion, and motivates them to invest more resources in their work. By providing long-term, clear, and attractive goals, the MV provides frontline service employees with sufficient psychological resources. This motivates them to exhibit positive service attitudes, invest more enthusiasm and initiative in customer service, and stimulate PCSP. Thus, this study proposes Hypothesis 2:

**H2.**  *The MV of frontline service employees plays a mediating role in the relationship between HCHRS and PCSP*.

### 2.3. The Mediating Role of Work Meaning

With many new generations of employees entering the labor market, employee WM has received unprecedented attention from researchers (Du & Mao, 2022; M. Song et al., 2018). Current research mainly adopts a dynamic perspective to examine WM. Lips-Wiersma and Wright (2012) and Steger et al. (2012) successively proposed that WM is an individual’s subjective experience of their work’s existence, meaning, and goal. Based on qualitative research, Bailey and Madden (2016) stated that WM arises when an individual feels an authentic connection between work and the individual’s broader, transcendent life goals. This study defines WM as the personal, subjective experience of frontline service employees concerning the existential meaning and purpose of their service work, reflecting their inner state.

The HCHRS has been found to increase employees’ job satisfaction by making them feel valued by the organization (Latorre et al., 2016). According to COR, the HCHRS provides employees with rich and comprehensive organizational resources through careful recruitment to ensure the fit between employees and the organization. By emphasizing overarching goals and teamwork, employees integrate goals that transcend the individual and have more positive judgments about the existential value of their service work, which also enriches their psychological resources. Through job rotation and employee participation in decision-making, employees are given richer work content and higher autonomy. They are motivated by appraisal based on effort, process, and competitive salary, so their external resources are enriched. Employees perceive that their rich work resources come from the comprehensive support of the organization. Therefore, their subjective service work experience is more positive, their enthusiasm for work is higher, and their sense of WM is enhanced.

Individuals with a high WM have been found to be less likely to experience emotional exhaustion (Lips-Wiersma & Wright, 2012) and have higher extra-role performance (Johnson & Jiang, 2017) with more proactive behaviors (Z. Ji & Xu, 2023). According to the COR, frontline service employees perceive the value of service work more positively, experience richer psychological and emotional resources, and can maintain a fuller state of mind. This, in turn, increases their enthusiasm for PCSP when performing emotional labor, such as service work. Thus, this study proposes Hypothesis 3:

**H3.**  *The WM of frontline service employees plays a mediating role in the relationship between the HCHRS and PCSP*.

### 2.4. Serial Mediation of Mission Valence and Work Meaning

The mediating roles of MV and WM are not simply parallel. It has been found that individual–organizational fit and a high-mission work orientation contribute to employees’ WM (Schnell et al., 2013; Wrzesniewski et al., 1997). Moreover, an increase in MV strengthens individuals’ intrinsic perceptions of the importance of their work (Guerrero & Chênevert, 2021). According to COR, when individual goals are more closely linked to the organizational mission, the organizational mission becomes an intrinsic motivation (Lin & Chen, 2018). This enriches employees’ psychological resources and creates a more positive perception of the existential value and subjective experience of their service work, which in turn enhances their WM.

According to SET, when employees perceive the work resources and trust given by the organization, they will also identify with and have an emotional attachment to the organization (Duan et al., 2017). HCHRS provides employees with rich work resources through systematic and multi-module measures. Following the principle of reciprocity, employees are more fully integrated into the organization’s mission by increasing recognition of the organization’s vision, enhancing the MV, and giving the organization positive feedback. According to COR, the psychological resources generated by MV provide intrinsic motivation and guide employees to perceive the important value of service work (Guerrero & Chênevert, 2021), further enhancing the work experience and strengthening WM. The enhancement of WM makes employees’ mental state more positive, which provides more sufficient emotional resources for employees’ emotional labor and ultimately stimulates PCSP.

By synthesizing the previous analysis, Hypothesis 4 can be proposed:

**H4.**  *The MV and WM of frontline service employees act as serial mediators of the influence of the HCHRS on PCSP*.

**Figure 1:**
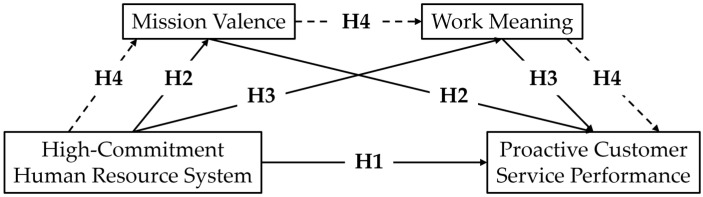
Theoretical framework.

Figure 1 demonstrates the theoretical framework of this study.

## 3. Methods

### 3.1. Measurement

The research scales for this study were developed with reference to mature scales validated by existing studies and widely accepted within the field (see Appendix A). The English-language scales were compiled strictly following the principle of “translation-back-translation” (Brislin, 1970). All scales were based on a 5-point Likert scale, with responses ranging from “strongly disagree” (1) and “strongly agree” (5).

Since the questionnaire survey was conducted in China, the High-Commitment Work System (HCWS) scale developed by (Xiao & Björkman, 2006) based on the Chinese context was chosen for the measurement of HCHRS. This scale involves the aspects of recruitment and selection, training, performance appraisal, job rotation, teamwork, remuneration and incentives, and intra-organizational communication. It also embodies the collectivist characteristic of Chinese culture, such as the “Appraisal of team performance rather than individual performance”. The scale has the advantages of covering more dimensions and being more adapted to the Chinese context, with good reliability and validity (Cronbach’s α = 0.77).

To measure MV, a 4-item scale that combines the perspectives of both individual emotions and organizational work importance was selected with reference to existing research (Pandey et al., 2008). The scale measures both the perceptual and rational aspects of employees’ perceptions of an organization’s mission with good reliability and validity. Its validity has been verified several times in MV studies (Cronbach’s α= 0.89; Yu & Zhang, 2023).

On the measurement of WM, this study selected the Work as Meaning Inventory (WAMI) scale developed by Steger et al. (2012). The scale categorizes WM into 10 items across three aspects: positive meaning, meaning-making through work, and greater good motivation. It is moderately itemized and demonstrates good reliability and validity (Cronbach’s α = 0.91).

For PCSP, the 7-item scale developed by Rank et al. (2007), which has been used in most studies, was chosen as the only widely used and validated instrument for measuring PCSP (Cronbach’s α = 0.77; H. Zhang et al., 2018).

Regarding control variables, this study drew on existing research and selected demographic variables such as gender, age, education, marital status, years of entry into the current organization, size of the organization, and years of establishment of the organization as control variables.

### 3.2. Samples

Data were collected using a questionnaire distributed in the form of paired questionnaires to frontline service employees and supervisors, who filled out separate items to avoid common method bias (CMB). The questionnaire consisted of two parts. The first part of the employee questionnaire consisted of scale items on MV, WM, and PCSP. The second part consisted of demographic information, including gender, age, education, marital status, and year of entry into the current organization. The first part of the supervisors’ questionnaire consisted of scale items for the HCHRS. The second part consisted of organization-related information, including the organization’s size and year of establishment. In the first part of the two questionnaires, each item was measured using a five-point Likert scale, with numbers “1–5” indicating the degree of conformity of the item.

Questionnaires were distributed in China from January to July 2024, both online and offline, using electronic questionnaires. To ensure the quality of the collected questionnaires, a paid form was adopted. Personnel engaged in frontline service work and their supervisors were invited to complete them. The collection of questionnaires was completely random, with the abbreviation of the organization or department name as the basis for pairing, and a comprehensive judgment was made based on the submitter’s IP address and submission time. If necessary, the corresponding respondents were asked to ensure the correct match.

The online questionnaire collection process is as follows. First, the questionnaire information was published on the online social platform to recruit survey subjects, conduct preliminary communication with volunteers who were willing to participate in the questionnaire filling, confirm that the respondents were engaged in customer service work, and confirm whether subordinates or leaders who can fill in the pairing questionnaire together were available. Second, they were informed of the precautions for filling in the pairing information, and the content and anonymity were clearly explained and emphasized. Respondents were instructed to answer the survey based on their actual situation and true feelings. After obtaining informed consent, the questionnaire filling could begin, and the respondents could withdraw at any time. Finally, after a preliminary check of the questionnaire, cash rewards were issued to the respondents. In addition, we proactively contacted frontline service employees in Jiangsu, Guangdong, Henan, and other locations to distribute offline questionnaires. Except for the different communication channels, the other processes are the same as above.

Ultimately, a total of 425 questionnaires were distributed in 26 provinces in China, and 331 valid questionnaires were obtained after removing invalid questionnaires, with a valid questionnaire rate of 77.88%. Table 1 presents the results for the control variables.

## 4. Results

### 4.1. Common Method Bias

To overcome the problem of CMB, this study emphasized anonymity and the purpose of the survey during the questionnaire collection process. Employee–supervisor pairings were used to collect the questionnaires, spreading out the scope and time of the research as much as possible to control CMB. After questionnaire collection, Harman’s single-factor test was conducted, which showed that the variance explained by the first factor before rotation was 36.86%, below the threshold of 40%. Additionally, to further test whether there was a problem of multicollinearity between the variables, a variance inflation factor test was conducted. The variance inflation factor (VIF) values were between 1.223 and 4.013, all below the threshold of 5. In summary, there was no serious CMB in this study.

### 4.2. Confirmatory Factor Analysis (CFA)

A CFA was performed on variables using AMOS26 to examine the discriminant validity between the four variables: HCHRS, MV, PCSP, and WM. Table 2 shows that the four-factor model has the best fit (χ^2^/*df* = 1.862, IFI = 0.912, TLI = 0.904, CFI = 0.911, RMSEA = 0.058, SRMR = 0.051) and is significantly better than the other models, indicating that there is good discriminant validity among the variables.

### 4.3. Correlation Analysis

As shown in Table 3, HCHRS is significantly and positively related to PCSP, significantly and positively related to MV, and significantly and positively related to WM. MV is significantly and positively related to PCSP and WM. WM is significantly and positively related to PCSP. In summary, we found significant correlations among all four variables, which provided preliminary support for the subsequent hypothesis testing. Further hypothesis testing was conducted to validate these findings.

### 4.4. Hypothesis Testing

A preliminary test of the hypotheses was performed using hierarchical multiple regression analysis. Path coefficients are expressed as standardized effect estimates (β) (Kline, 2016).

Firstly, we verified the effect of HCHRS on MV, PCSP, and WM. As shown in Table 4, Model 1, HCHRS has a significant positive effect on MV (β = 0.70, *p* < 0.01); as shown in Model 2, HCHRS has a significant positive effect on WM (β = 0.69, *p* < 0.01); as shown in Model 5, HCHRS has a significant positive effect on PCSP (β = 0.71, *p* < 0.01).

Second, the mediating roles of the MV and WM were verified. According to Model 6, HCHRS (β = 0.34, *p* < 0.01) and MV (β = 0.53, *p* < 0.01) simultaneously play a significant positive effect on PCSP. According to Model 7, HCHRS (β = 0.32, *p* < 0.01) and WM (β = 0.56, *p* < 0.01) simultaneously played a significant positive role on PCSP.

Finally, the serial mediating roles of MV and WM were verified. As shown in Model 8, HCHRS (β = 0.28, *p* < 0.01), MV (β = 0.25, *p* < 0.01), and WM (β = 0.37, *p* < 0.01) positively and significantly influence PCSP.

However, the hierarchical multiple regression results cannot reflect the measurement error. To further verify the mediation effect and ensure the robustness and rigor of the hypotheses results, we also used AMOS 28 to build a structural equation model and performed 5000 bootstrap tests. Path coefficients are expressed as standardized effect estimates (β) (Kline, 2016). Additionally, both percentile (PC) and bias-corrected (BC) methods are used to calculate confidence intervals.

**Figure 2:**
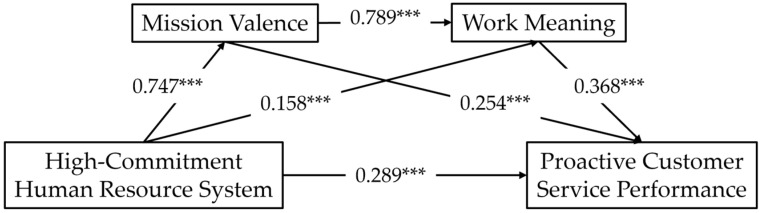
Results of bootstrap tests. Note: *** means *p* < 0.001.

According to Figure 2, HCHRS has a positive and significant effect on MV (β = 0.747, *p* < 0.001), WM (β = 0.158, *p* < 0.001), and PCSP (β = 0.289, *p* < 0.001). MV has a significant positive effect on the sense of WM (β = 0.789, *p* < 0.001) and PCSP (β = 0.254, *p* < 0.001). WM has a significant positive effect on PCSP (β = 0. 368, *p* < 0.001); H1 is validated.

In Table 5, the mediating effect value of MV is 0.224 (*p* = 0.002), that of WM is 0.069 (*p* < 0.001), and the serial mediation effect value is 0.256 (*p* < 0.001). Under both PC and BC methods, the direct (β = 0.289, *p* < 0.001), total (β = 0.754, *p* < 0.001), and three indirect effects mediating paths do not include 0 at the 95% confidence interval. This proves that MV and WM both play the role of partial and serial mediators in influencing the PCSP of the HCHRS, and H2, H3, and H4 are validated.

## 5. Discussion

First, this study discussed the direct mechanisms by which the HCHRS influences PCSP. Based on SDT, the HCHRS positively influences PCSP by satisfying employees’ needs for job autonomy, perceived competence, and relationship needs. This is achieved through multiple measures, including enhancing employee ability and providing more opportunities, such as investing in employees through comprehensive training, job rotation arrangements, and internal recruitment for managerial positions. Furthermore, this also satisfies employees’ belonging needs and improves their motivation through people-oriented management, such as strengthening inter-level communication, enriching workplace life, and providing competitive remuneration. This finding is supported by the existing literature (M. Chen et al., 2017).

Second, we found a mediating effect of MV on the effect of the HCHRS on PCSP. Combining the perspectives of SET and COR, the HCHRS creates an environment of mutual benefit between the organization and the individual. Institutionalized and systematic organizational support is a reflection of the organization’s fulfillment of social responsibilities. An organization’s goodwill toward employees encourages them to prioritize its mission and vision in their work and identify with and integrate into such a mission. This enhances the MV of the employees and enriches psychological resources, thereby increasing PCSP. This finding echoes those reported by Yuan et al. (2022).

Finally, this study explored the mediating role of WM among the mechanisms by which the HCHRS influences PCSP based on the COR. The HCHRS provides comprehensive support to frontline service employees, enriches their work resources including external and psychological resources as well as other forms of substantial organizational support, and improves their service work experiences. In other words, the HCHRS builds a more complete talent growth system, and employees have higher recognition of the value of their service work, ultimately improving their WM. As an internal state, WM enriches employees’ psychological and emotional resources. Therefore, employees’ feelings about the meaning of work can be transformed into motivation for service work, which motivates them to implement more PCSP.

Additionally, according to COR and SET, a correlation also exists between MV and WM. An improvement in MV is a positive piece of feedback to the organization regarding the implementation of an HCHRS; it strengthens the individual’s perception of the importance of the organization’s mission and further enhances their intrinsic perception of the importance of the work (Guerrero & Chênevert, 2021). This provides richer psychological resources for employees to devote themselves to service work, creates a more positive work experience, and positively affects WM. A high WM enriches employees’ psychological resources (Lips-Wiersma & Wright, 2012), which ultimately increases the implementation of PCSP. This finding emphasizes the mutual nature of the organization–employee relationship.

## 6. Conclusions

This study constructs a serial mediation model, discusses the influence mechanism of the HCHRS on PCSP, and draws several conclusions. The HCHRS enables organizations to establish a positive long-term connection with employees, positively and significantly influencing the PCSP of frontline service employees. MV, as a repayment for organizational goodwill, plays a mediating role in the influence of the HCHRS on PCSP. WM, as a result of the improved work experience of the HCHRS, makes employees more enthusiastic about service work and play a mediating role in the influence of the HCHRS on PCSP. MV and WM played serial mediating roles in the influence of HCHRS on PCSP. The research findings deepen the discussion on the relationship between HCHRS and PCSP from an organizational perspective, thus broadening the boundaries of research on the factors influencing PCSP.

## 7. Practical Implications

Service quality is a fundamental and sustainable competitive advantage for service organizations. It is significant for cultivating new productive forces and achieving high-quality development within the service industry. Faced with an increasingly uncertain environment, stimulating frontline service employees’ PCSP plays an important role in improving service quality, increasing customer satisfaction, and enhancing organizational competitiveness. Without satisfied employees, there are no satisfied customers. In the face of today’s frontline service employees, who attach more importance to comprehensive work experience, organizations need to optimize various aspects of HR systems.

The HCHRS is a comprehensive enhancement of employee competence, sense of identity, and sense of mission within the human resource management mode. The HCHRS can stimulate employees’ enthusiasm to take the initiative to serve and is a reference direction for human resource management reform in service-oriented organizations. The organization can provide more learning opportunities for frontline service employees, carry out consumer behavior knowledge training and service experience exchange meetings, and provide various job rotation opportunities to enhance employees’ comprehensive ability. Through a competitive salary system and employee development-oriented performance appraisal, employees perceive organizational support. This motivates them to repay the organization and enhances their enthusiasm for providing excellent service. Strengthening information sharing within the organization, granting more authorization to frontline service employees, and establishing a regular communication mechanism between frontline service employees and management breaks down barriers to implementing PCSP. This ultimately motivates more employees to embrace and implement PCSP.

Employees with a high MV were more willing to perform PCSP. Organizations recruiting frontline service personnel can focus on the degree of recognition of the organization’s mission and focus on candidates who have a higher understanding of corporate values and mission vision. For existing employees in the organization, while strengthening the interpretation of mission and vision, organizations should also consider the general demands of employees. This helps determine a mission and vision that is more attractive to employees, thereby improving the attractiveness of the organization’s values and mission vision. This approach encourages employees to actively integrate into the organization’s mission and stimulate PCSP.

Employees with high WM tend to be more enthusiastic about implementing extra-role behaviors. Organizations can improve the leadership style of service supervisors through leadership training, shape high-quality leadership–employee relationships, enhance employees’ work experience, and then enhance employees’ WM. Moreover, through the development of a transparent system of work rights and responsibilities, the assessment focuses on team performance rather than individual performance and carries out a variety of group-building activities to enhance team cohesion, promote teamwork, and strengthen the positive relationship between the organization and employees. This improves the WM and ultimately encourages employees to interact actively with customers and colleagues through PCSP.

## 8. Limitations and Prospects

First, regarding the research design, the sample source of this study is primarily the entity service industry, and it paid insufficient attention to the group of digital platform service workers, which has been growing in recent years. Future research can refer to the studies of Pei et al. (2022) and Kan et al. (2024), which consider digital platform service workers as the primary source of the sample, making the findings more contemporary. Although this study chose employee–supervisor pairings for questionnaire collection and passed Harman’s single-factor test and VIF test without serious CMB, it did not adopt the method of multi-time-point questionnaire collection due to the research conditions. Future research could consider using the method of multi-time-point collection to further reduce the possibility of CMB.

Second, regarding the theoretical framework, this study constructed a complete and comprehensive serial mediation model. However, future research could still try to introduce moderating variables and construct a moderated mediation model to explore the mechanism of the influence of HCHRS on PCSP. As more stable and difficult-to-change characteristics, certain personality traits make employees more likely to engage in PCSP in the same environment, and studies have verified the moderating effect of traits such as emotional intelligence, mindfulness, and proactive personality on PCSP (Huo et al., 2019; Yan et al., 2023). Future research could further explore other moderating variables affecting PCSP, construct a mediation model with moderation, and enrich our understanding of the formation mechanism of PCSP.

Finally, although this study expands the research boundaries of the factors influencing PCSP, it does not discuss its influence owing to the research conditions. Existing studies have paid little attention to the influence of PCSP (H. Zhang et al., 2018). Some studies have verified the positive effect of PCSP on customer satisfaction (Raub & Liao, 2012) and customer engagement (Siami et al., 2022). At the same time, Wang et al. (2021) pointed out that PCSP does not always lead to a good experience for the customer and that if an employee misjudges the customer’s real needs, implementing PCSP may be counterproductive. Additionally, there have been no other discussions on the impact of PCSP. If employees perform PCSP and receive appreciation or gratitude from customers, will their WM increase? Are employees willing to perform more PCSP? Can there be a virtuous circle between WM and PCSP? Owing to the fragility of WM (Bailey & Madden, 2016), will employees who perform PCSP but do not receive positive feedback experience a decrease in WM or even emotional exhaustion? Future studies could focus on these issues.

In addition, this study focused on the Chinese context. Although the scope of the questionnaire survey was as broad as possible, future research can further improve the generalizability of the research conclusions by investigating multiple contextual backgrounds. Future research can focus on cross-cultural diverse contexts to explore the impact of the HCHRS on employees in different cultural backgrounds, as well as the path for frontline service personnel in different national conditions to form PCSP. For example, power distance is a classic concept for measuring cultural differences (Hofstede, 1980). Employees with high power distance may rely more on superior instructions rather than autonomous decision-making. Employees with low power distance may welcome an HCHRS that promotes equality, but they may be less willing to follow organizational guidance. Will these factors affect the psychology of frontline service employees in different contextual backgrounds, thereby regulating the impact of the HCHRS on PCSP? Similar questions can be explored in future research.

## Figures and Tables

**Table 1:** (a) Results of the control variables. (b) Results of the control variables.

**(a)**		
**Variable**	**Form**	**Percentage (%)**
gender	Male	45.6
	Female	54.4
age	24 and below	10.6
	25–29	32.3
	30–34	31.7
	35–39	17.2
	40–44	3.9
	45 and above	4.2
education	junior high school and below	2.1
	high school/technical secondary school	7.3
	junior college	11.8
	Bachelor’s degree	58.6
	Master’s degree and above	20.2
marital status	unmarried	39.0
	married	59.5
	divorced	1.5
year of entry into the organization	1 year and shorter	11.5
	1–3	27.2
	3–5	47.1
	5–7	11.2
	8–10	1.8
	10 and longer	1.2
**(b)**		
**Variable**	**Form**	**Percentage (%)**
size of the organization	Less than 10 people, or annual operating income of less than CNY 1 million	2.1
	10 to 100 people, or an annual operating income of CNY 1 million to 20 million	16.3
	100 to 300 people, or an annual operating income of CNY 20 million to 100 million	68.0
	More than 300 people, or an annual operating income of more than CNY 100 million	13.6
year of establishment of the organization	1 year and shorter	0.3
	1–3	5.1
	3–5	14.8
	5–7	12.7
	8–10	45.3
	10 years and longer	21.8

**Table 2:** CFA results.

Model	χ^2^	*df*	χ^2^/*df*	IFI	TLI	CFI	RMSEA	SRMR
four-factor model (HCHRS/MV/WM/PCSP)	1085.336	583	1.862	0.912	0.904	0.911	0.051	0.051
three-factor model (HCHRS/MV + WM/PCSP)	1250.412	591	2.116	0.885	0.876	0.884	0.058	0.056
two-factor model (HCHRS/MV +WM + PCSP)	1268.371	593	2.139	0.882	0.874	0.881	0.059	0.057
single-factor model (HCHRS + MV +WM + PCSP)	1330.115	594	2.239	0.871	0.862	0.870	0.061	0.057

Note: HCHRS stands for high-commitment human resources system, MV stands for mission valence, WM stands for work meaning, and PCSP stands for proactive customer service performance.

**Table 3:** Results of correlation analysis.

Variable Name	1	2	3	4	5	6	7	8	9	10	11
1. HCHRS	1										
2. MV	0.747 ***	1									
3. WM	0.754 ***	0.804 ***	1								
4. PCSP	0.748 ***	0.907 ***	0.815 ***	1							
5. gender	−0.159 **	−0.244 ***	−0.184 **	−0.285 ***	1						
6. age	−0.145 **	−0.179 **	−0.229 ***	−0.217 ***	0.280 ***	1					
7. education	0.217 ***	0.208 ***	0.229 ***	0.177 **	0.146 **	−0.224 ***	1				
8. marital status	−0.148 **	−0.209 ***	−0.230 ***	−0.245 ***	0.229 ***	0.675 ***	−0.062	1			
9. year of entry into the organization	−0.001	−0.006	−0.041	−0.027	0.127 *	0.503 ***	−0.007	0.530 ***	1		
10. year of establishment of the organization	0.042	0.062	0.069	0.056	−0.070	0.048	−0.108 *	0.051	0.071	1	
11. size of the organization	−0.111 **	−0.012	−0.044	−0.075	0.025	−0.031	0.167 **	−0.035	−0.053	0.234 ***	1
Mean	3.685	3.606	3.556	3.845	1.540	2.840	3.880	1.630	2.680	4.630	2.930
SD	0.563	0.984	0.867	0.663	0.499	1.198	0.887	0.515	0.962	1.146	0.616

Note: * means *p* < 0.05, ** means *p* < 0.01, *** means *p* < 0.001; HCHRS stands for high-commitment human resources system, MV stands for mission valence, WM stands for work meaning, and PCSP stands for proactive customer service performance.

**Table 4:** Results of hierarchical multiple regression.

Variable Name	MV	WM			PCSP			
	Model 1	Model 2	Model 3	Model 4	Model 5	Model 6	Model 7	Model 8
HCHRS	0.703 ***	0.690 ***		0.150 ***	0.709 ***	0.336 ***	0.321 ***	0.282 ***
MV			0.877 ***	0.767 ***		0.530 ***		0.249 ***
WM							0.562 ***	0.366 ***
gender	−0.129 **	−0.155 ***	−0.054 *	−0.056 *	−0.046	0.023	0.042	0.043
age	0.023	−0.006	−0.024	−0.024	−0.057	−0.069	−0.054	−0.061
education	0.066	0.045	0.008	−0.005	0.066	0.031	0.040	0.033
marital status	−0.120 *	−0.138 **	−0.045	−0.046	−0.097	−0.033	−0.019	−0.017
year of entry in the organization	0.065	0.068	0.017	0.018	0.043	0.009	0.005	0.002
year of establishment of the organization	−0.019	0.026	0.017	0.012	0.045	0.035	0.031	0.031
size of the organization	0.054	−0.011	−0.071 **	−0.052 *	0.011	−0.018	0.017	0.001
R^2^	0.582	0.593	0.830	0.839	0.585	0.701	0.712	0.722
ΔR^2^	0.430	0.413	0.644	0.653	0.437	0.552	0.563	0.573

Note: * means *p* < 0.05, ** means *p* < 0.01, *** means *p* < 0.001; HCHRS stands for high-commitment human resources system, MV stands for mission valence, WM stands for work meaning, and PCSP stands for proactive customer service performance.

**Table 5:** Results of the mediation effect test.

Path	β	se	*p*	95% CI (PC)		95% CI (BC)	
				LLCL	LLCL	ULCL	ULCL
HCHRS→PCSP (direct effect)	0.289	0.062	<0.001	0.222	0.462	0.220	0.461
HCHRS→MV→PCSP	0.224	0.074	0.002	0.082	0.373	0.089	0.381
HCHRS→WM→PCSP	0.069	0.023	<0.001	0.028	0.120	0.030	0.124
HCHRS→MV→WM→PCSP	0.256	0.059	<0.001	0.138	0.373	0.138	0.374
total effect	0.754	0.051	<0.001	0.786	0.989	0.783	0.986

Note: HCHRS stands for high-commitment human resources system, MV stands for mission valence, WM stands for work meaning, and PCSP stands for proactive customer service performance.

## Data Availability

The data used to support the findings of this study are available from the corresponding author upon request.

## Appendix A

The 7 items of Proactive Customer Service Performance Scale (Rank et al., 2007)
  1. I proactively share information with customers to meet their financial needs.
  2. I anticipate issues or needs customers might have and proactively develops solutions.
  3. I use own judgment and understanding of risk to determine when to make exceptions or improvise solutions.
  4. I take ownership by following through with the customer interaction and ensures a smooth transition to other service representatives.
  5. I actively create partnerships with other service representatives to better serve customers.
  6. I take initiative to communicate client requirements to other service areas and collaborates in implementing solutions.
  7. I proactively check with customers to verify that customer expectations have been met or exceeded.
The 4 items of Mission Valence Scale (Pandey et al., 2008)
  1. This organization provides valuable public services.
  2. I believe that the priorities of this organization are quite important.
  3. The work of this organization is not very significant in the broader scheme of things.
  4. For me, the mission of this organization is exciting.
The 10 items of Work Meaning Scale (Steger et al., 2012)
  1. I have found a meaningful career.
  2. I view my work as contributing to my personal growth.
  3. My work really makes no difference to the world.
  4. I understand how my work contributes to my life’s meaning.
  5. I have a good sense of what makes my job meaningful.
  6. I know my work makes a positive difference in the world.
  7. My work helps me better understand myself.
  8. I have discovered work that has a satisfying purpose.
  9. My work helps me make sense of the world around me.
  10. The work I do serves a greater purpose.
The 15 items of High-Commitment Human Resource System Scale (Xiao & Björkman, 2006)
  1. Promotion from within rather than from outside.
  2. Careful selection procedures in recruiting.
  3. Extensive training and socialization.
  4. Trying not to fire employees.
  5. Enlarged jobs and job rotation.
  6. Appraisal of team performance rather than individual performance.
  7. Attitude and behaviour-oriented appraisal rather than result-oriented appraisal.
  8. Feedback for development purposes rather than for evaluation purposes.
  9. High remuneration, including compensation and fringe benefits.
  10. Extensive ownership of shares, options or profit-sharing.
  11. Trying to promote egalitarianism in income, status and culture.
  12. Participation in forms of suggestion, grievance systems and morale survey.
  13. Open communication and wide information sharing.
  14. Emphasizing strong overarching goals.
  15. Work in teams; successes of teams rather than individual are hailed.
